# Outbreak of Chikungunya Virus Infection, Vanimo, Papua New Guinea

**DOI:** 10.3201/eid1909.130130

**Published:** 2013-09

**Authors:** Paul F. Horwood, Lisa J. Reimer, Rosheila Dagina, Melinda Susapu, Grace Bande, Michelle Katusele, Gussy Koimbu, Stella Jimmy, Berry Ropa, Peter M. Siba, Boris I. Pavlin

**Affiliations:** Papua New Guinea Institute of Medical Research, Goroka, Papua New Guinea (P.F. Horwood, G. Bande, P.M. Siba);; Papua New Guinea Institute of Medical Research, Madang, Papua New Guinea (L.J. Reimer, M. Katusele, G. Koimbu);; National Department of Health, Port Moresby, Papua New Guinea (R. Dagina, M. Susapu, B. Ropa);; Vanimo General Hospital, Vanimo, Papua New Guinea (S. Jimmy);; World Health Organization, Port Moresby (B.I. Pavlin)

**Keywords:** chikungunya, chikungunya virus, CHIKV, viruses, mutant strain, Aedes albopictus, outbreak, Papua New Guinea

## Abstract

In June 2012, health authorities in Papua New Guinea detected an increase in febrile illnesses in Vanimo. Chikungunya virus of the Eastern/Central/Southern African genotype harboring the E1:A226V mutation was identified. This ongoing outbreak has spread to ≥8 other provinces and has had a harmful effect on public health.

Chikungunya virus (CHIKV) is a mosquito-transmitted virus of the family *Togaviridae* and genus *Alphavirus*. CHIKV can be classified into 3 distinct genotypes: Asian, Eastern/Central/Southern African (ECSA), and Western African. The usual vectors for CHIKV are *Aedes aegypti* mosquitoes; *Ae. albopictus* mosquitoes are a potential secondary vector. Human infection with CHIKV results in illness characterized by high fever, severe polyarthralgia, headache, maculopapular rash, fatigue, nausea, and vomiting. CHIKV has recently been responsible for explosive outbreaks of disease in the Indian Ocean region (*1*) and southern India (*2*).

Before June 2012, chikungunya had not been reported in Papua New Guinea. The recent increase in reported outbreaks of chikungunya in Papua New Guinea has coincided with the appearance of a variant strain of CHIKV that has a mutation from alanine to valine at amino acid position 226 in the envelope 1 (E1) glycoprotein gene. This mutation enables CHIKV strains to more efficiently replicate in the salivary gland of *Ae. albopictus* mosquitoes, thus enhancing the role of this vector in transmission of virus to susceptible human hosts (*3*). We report molecular detection, epidemiologic and entomologic investigations, and viral genetic characterization for an outbreak of chikungunya in Papua New Guinea.

## The Study

In late June 2012, an increase in cases of prolonged fever for ≥3 days was reported from the Vanimo General Hospital in Vanimo, Sandaun Province. The illness was characterized by high fever (temperature >40°C), arthralgia, emesis, and severe nausea. In most patients, fever subsided within 24–72 hours and patients were discharged after abatement of initial signs and symptoms. However, many patients returned within a few days reporting lingering arthralgia and severe pruritus. On the basis of clinical characteristics, several arboviruses were immediately suspected as causes of the illness.

**Figure 1:**
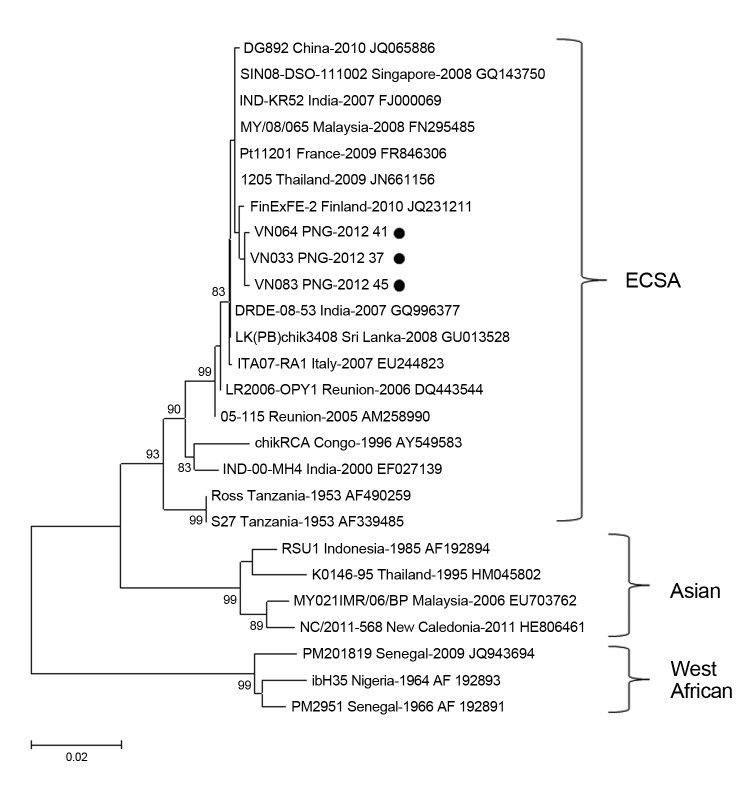
Phylogenetic analysis of a 924-nt fragment of partial envelope 1 glycoprotein gene sequences of chikungunya virus strains isolated in Vanino, Papua New Guinea (PNG) and global strains. The tree was constructed by using bootstrap analysis (1,000 replicates) and the neighbor-joining method with the Kimura 2-parameter method for nucleotide data analysis. Values along branches are bootstrap percentages. Black circles indicate strains isolated in this study. ECSA, Eastern/Central/Southern African. Scale bar indicates nucleotide substitutions per site.

Serum samples were collected from 86 patients with acute fever during September–October 2012. Samples were screened for CHIKV by using a reported real-time reverse transcription PCR (*4*), and 31 (36%) were positive for CHIKV. Logistic regression showed that no clinical findings were predictive of laboratory confirmation of CHIKV. Partial E1 genes for 3 CHIKV strains from Papua New Guinea (VN033–KC524770, VN064–KC524771, and VN083–KC524772) were sequenced (*4*). The Papua New Guinea strains had high levels of nucleotide (99.9%) and amino acid (100%) identity; only 2 synonymous sequence polymorphisms were observed between the 3 strains. Sequence alignment and phylogenetic analysis of E1 sequences showed that the Papua New Guinea outbreak was caused by CHIKV from the ECSA genotype (Figure 1). All 3 CHIKV strains had the E1:A226V mutation.

**Figure 2:**
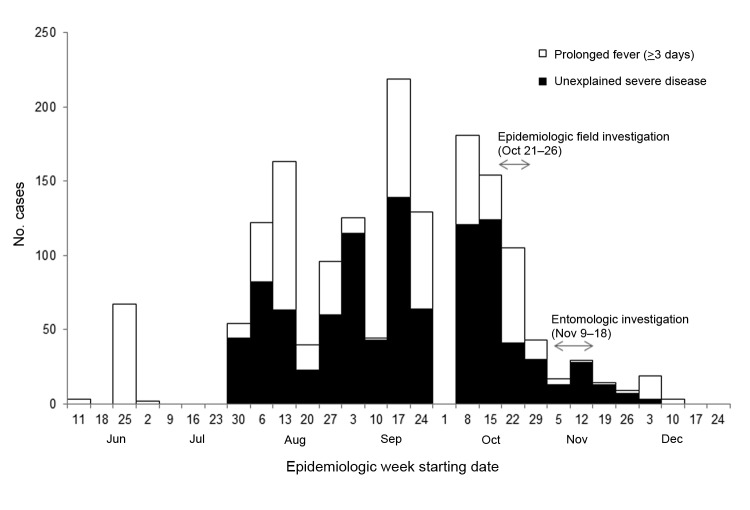
Syndromic cases (n = 1,565) reported from Vanimo General Hospital, Papua New Guinea, July–October 2012. Two of the 8 syndromes under surveillance were increased during the chikungunya outbreak. It is not known why no cases were recorded during the week of October 1.

During June 25–November 25, a total of 1,590 suspected cases of chikungunya were recorded at Vanimo General Hospital (Figure 2). Detailed data were collected for 98 patients (54 female patients; p = 0.31). The age range of the patients was 2–60 years (median age 24 years). The most common signs and symptoms were fever and arthralgia (100%; these symptoms constituted the case definition), headache (83%), cough (31%), and nausea (26%).

In November 2012, an entomologic survey was conducted at 49 households in Vanimo and surrounding settlements. All potential indoor and outdoor breeding containers were recorded and container, household, and Breteau indices were calculated (Table) on the basis of the presence of aedine larvae. A portion of larvae from each collection was reared to adults for morphologic identification, and 100% (n = 137) were identified as *Ae. albopictus* mosquitoes.

**Table:** Descriptive data for entomologic survey for chikungunya virus, Vanino, Papua New Guinea

Characteristic	Value
No. houses surveyed	49
No. containers with water	898
No. mosquito-positive containers	159
No. mosquito-positive outdoor containers (%)	157 (99)
No. mosquito-positive refuse containers (%)	117 (74)
House index, % (range)	76 (43–100)
Container index, % (range)	18 (10–50)
Breteau index (range)	324 (150–729)

Adult mosquitoes were collected over 10 days by using the Biogents-Sentinel odor-baited trap (Biogents AG, Regensberg, Germany). All 155 mosquitoes collected were identified morphologically to species; 154 were identified as *Ae. albopictus* mosquitoes, and 1 was identified as an *Ae. scutellaris* mosquito. Mosquitoes were separated by sex and location and combined in pools of ≤10 for detection of CHIKV by use of real-time reverse transcription PCR. All mosquito pools were negative for CHIKV.

## Conclusions

The outbreak of chikungunya in Vanimo is still ongoing. There are grave concerns that the outbreak will spread throughout the country; as of this writing, confirmed or suspected cases have been reported in 8 other provinces (Madang, West New Britain, East New Britain, New Ireland, Eastern Highlands, Oro, Chimbu, and Morobe) in Papua New Guinea.

Although no previous outbreaks of chikungunya in Papua New Guinea have been documented, a study conducted in the mid-1970s reported that the seroprevalence of CHIKV was ≤30% in some regions of the country (*5*). A similar study at that time also reported a high seroprevalence of CHIKV throughout Indonesia and Southeast Asia (*6*). These studies suggest that there may have been a widespread outbreak of chikungunya in the region during preceding years. However, the antigenic cross-reactivity of arboviruses is widely recognized and cannot be ruled out as a cause of the high seropositivity to CHIKV. The broad age distribution of persons in the outbreak reported here suggests a lack of neutralizing antibodies against CHIKV, at least in the Vanimo area.

The recent evolution of ECSA strains with the E1:A226V mutation has resulted in several outbreaks of chikungunya in widely distributed geographic regions. The emergence of these strains seems to be a case of convergent evolution with adaption to *Ae. albopictus* mosquitoes occurring during outbreaks in India and the Indian Ocean region (*7*). Analysis of E1 genes in this study suggests that virus strains in Papua New Guinea are closely clustered with mutant strains that evolved in India and subsequently spread to other countries, such as Malaysia, Singapore, Sri Lanka, Thailand, and Italy. However, full-genome analysis is required to establish the relationship between strains from Papua New Guinea and other regions.

Neighborhoods in Vanimo had a high Breteau index of 324, which indicated high transmission potential for CHIKV (*8*) and dengue virus (*9*). *Ae. albopictus* mosquitoes were found breeding in many types of containers found outdoors and in vases found indoors. Most infested containers were water storage vessels, such as old metal drums and buckets without proper coverings; in some instances, covered vessels were also infested. Old tires were a highly used breeding site, and gutters were observed to be densely populated.

Because of the absence of a reticulated water system in Vanimo and the general ubiquity of artificial containers (water storage and refuse), our observations indicated that opportunities were rife for breeding of *Aedes* spp. mosquitoes. These breeding opportunities existed despite the fact that Sandaun Provincial Health authorities initiated multiple cleanup campaigns before our survey (although no other vector control measures were implemented).

Because of logistic constraints, the entomologic survey was conducted during the decrease in the outbreak and was designed to identify the dominant vector and opportunities for control. We were unable to positively implicate *Ae. albopictus* mosquitoes as the primary vector. However, the predominance of this mosquito throughout the outbreak region (*10*,*11*) and its role in CHIKV transmission in other regions (*12*–*14*) highlights the need for targeted vector control to curtail the spread of disease.
